# Systematic data management for effective AI-driven decision support systems in robotic rehabilitation

**DOI:** 10.1038/s41598-025-09740-2

**Published:** 2025-07-30

**Authors:** Anastasios Tzepkenlis, Cristian Camardella, Marco Germanotta, Irene Giovanna Aprile, Maria Cristina Mauro, Daniele Leonardis, Antonio Frisoli

**Affiliations:** 1 Istituto di Intelligenza Meccanica, Scuola Superiore Sant’Anna, Via L. Alammanni 13b, 56010 Pisa, Italy; 2 IRCCS Fondazione Don Carlo Gnocchi, Via di Scandicci 269, 50143 Florence, Italy

**Keywords:** Robotics, Rehabilitation, Kinematics, Machine learning, Deep neural networks, Regression, Decision support system, Post-stroke, Computer science, Health care, Prognosis, Biomedical engineering

## Abstract

Robotic rehabilitation is becoming a standard in post-stroke physical rehabilitation, and these setups, often coupled with virtual exercises, collect a large and finely grained amount of data about patients’ motor performance, in terms of kinematics and force interactions. Given the high resolution of data throughout the rehabilitation treatment, invaluable information is concealed, especially if oriented towards

predictive systems and decision support systems. Nevertheless, a comprehensive understanding of how manipulating these datasets with machine-learning to produce such outputs is still missing. This study leverages comprehensive robotic-assisted rehabilitation data to systematically investigate clinical outcome predictions (FMA, ARAT and MI) and robot parameters suggestions based solely on kinematic and demographic data. Our method significantly outperforms conventional approaches on both tasks demonstrating the potential of systematic data handling in advancing rehabilitation practices. Moreover, under the explainable-AI policies, a focus on prediction power of variables and a clinical knowledge base of predicted outcome are provided.

## Introduction

In the era of artificial intelligence, the demand for advanced systems to enable personalized and effective rehabilitation treatments drives research to demonstrate their efficacy. Robotics rehabilitation setups are increasingly becoming popular in clinical settings, as they show to be effective in achieving better clinical outcomes on motor recovery^1–3^, also taking advantage of the virtual serious games approach, coupled with the robotic device, to foster patient engagement, commitment, and intensity of the exercises^4^. One of the advantages these system have, compared to conventional approaches, is the capability to record a wide amount of data throughout the rehabilitation process of patients. Data usually include kinematic information (e.g., position of the robot end-effector and/or joint angles) reflecting patients’ movement and interaction forces, task-specific information (e.g., the serious game status, scores, target positions, etc.), and, optionally, biometric and psychophysiological data. In literature, correlation analyses have been performed looking for potential links between rehabilitation instrumented data and clinical scale scores^5–7^ or to build predictive systems^8,9,44^. The goal was to provide healthcare professionals a tool to get insights about the ongoing rehabilitation process without the need to perform clinical assessments, which is notably a long lasting and costly operation. Particularly, predictive systems are increasingly valuable in medicine, offering tools to anticipate outcomes, and in turn use this information to improve resource allocation, optimize treatments, and enhance personalized care^10,11^. For instance, in post-stroke rehabilitation, these systems assist clinicians in navigating complex recovery processes, facilitating data-driven decisions that influence patient recovery pathways^12^.

The integration of these predictive models into Clinical Decision Support Systems (CDSS) represents a significant opportunity to enhance rehabilitation outcomes. By combining kinematic data with demographic factors such as age, sex, and latency (time since the first stroke), CDSSs can provide actionable insights into clinical outcomes and suggest personalized parameters for robotic-assisted exercises. This approach not only supports therapists in making data-driven decisions but also reduces bias in treatment delivery, ensuring a more standardized and effective rehabilitation process. Recent advancements in CDSSs, powered by AI techniques (rule-based, machine learning, and deep learning), have enabled faster, more accurate, and cost-effective decision-making^13^. These systems are designed to enhance medical decisions by integrating targeted clinical knowledge and patient data^14^.

In literature, most of the works on CDSSs and rehabilitation predictive systems focused on clinical and demographics based datasets to build long-term or short-term predictions on clinical scale scores^15,16^, mortality^17,18^, or functional motor recovery^19,20^.

A research gap in this topic is the appropriate usage of kinematic data in predictive models, with a method able to leverage on inherently complex time-dependent features. These datasets capture the dynamic nature of patient movements, enabling the extraction of meaningful patterns that can inform clinical decisions. The creation of structured time-series datasets from robotic setups is essential for exploiting information embedded in the rehabilitation output data. Development of a systematic approach is still uncovered in literature, and to address the gap, a deep understanding of patterns underlying collected data is needed. This is an insight that, nowadays, machine learning can provide with satisfying results. Neural networks, particularly those designed to handle sequential data, have shown exceptional performance in modeling such complex relationships, making them highly effective for clinical prediction problems^21,22^. For instance, they have been used to predict cardiovascular health trajectories using electronic health records^23^, classify diabetes patients through combined convolutional and LSTM approaches^24^, and forecast stroke risk using demographic and risk factor data^25^. Their ability to model temporal dependencies makes them particularly suited for applications in rehabilitation, where patient progress is tracked over time. In robotic-assisted rehabilitation, specific neural networks have been used to guide motor assistance based on healthy or therapist-performed trajectories^26^ and predict motor capability decline in Parkinson’s patients using kinematic data^27^. While explainability is often considered crucial for building clinician confidence in AI systems^28^, excessive reliance on AI reasoning explanations can lead to uncritical trust, potentially increasing medical errors and patient harm^29^. In the context of rehabilitation, where therapist’s experience play a vital role, it is essential to strike a balance between leveraging AI-driven insights and maintaining the therapist’s active involvement in decision-making. This approach ensures that AI systems complement, rather than replace, the critical judgment of healthcare professionals.

**Fig. 1:**
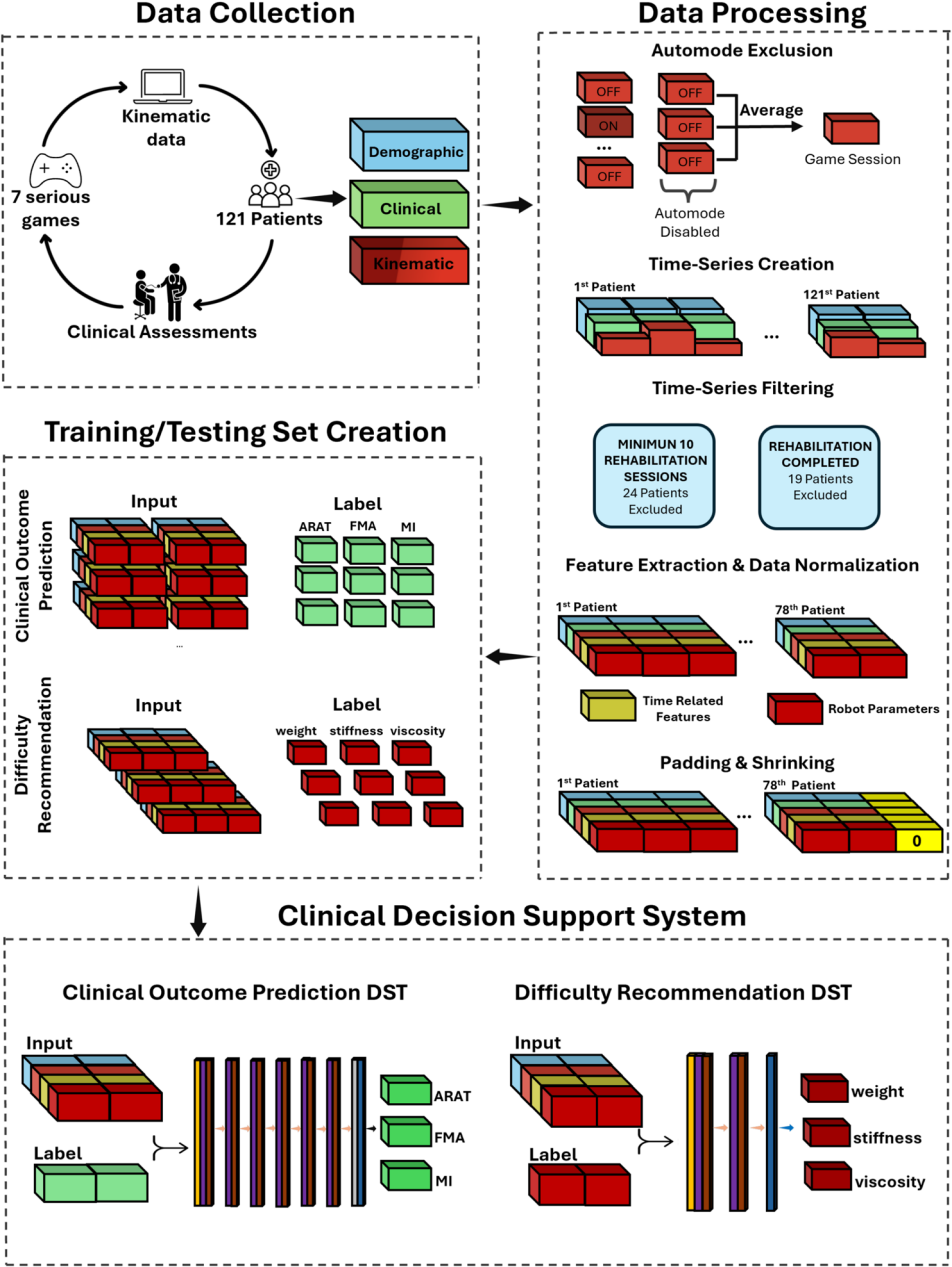
Illustration of the proposed pipeline for developing the Clinical Decision Support System. Data is collected either by the robot (kinematic data) or through an initial assessment (demographic and clinical data) or at discharge (clinical data) by therapists from 121 post-stroke patients. The collected data is preprocessed into structured data frames, which are then separated for training and testing the final models (the clinical outcome prediction model and the difficulty level recommendation model).

To fill the aforementioned research gap, this study presents a novel approach for kinematic data handling, including also demographic data, covering a typical dataset collected with robotic rehabilitation setups and serious games. This method has then been experimented with the processing of an LSTM model, with the aim of predicting the clinical scale scores at the end of rehabilitation in robotic-assisted neurorehabilitation, together with the prediction of the therapist’s recommendations on the exercise parameters. This experimental application leverages a substantial dataset from 121 post-stroke patients undergoing full robotic-assisted treatments with continuous data recording. The approach benefits from detailed session data and conventional clinical scale assessments, ensuring robust predictive capability and actionable insights Fig. 1.

Our main contributions are the following:

- We propose a systematic approach to effectively translate robotic-rehabilitation data into a time-series dataset, enabling the use of advanced neural networks for outcome prediction.
- We provide the prototype of a Clinical Decision Support System, based on our novel data handling method, that predicts clinical outcomes and recommends difficulty levels for each serious game-mediated exercise based on previously acquired human expert knowledge. This system supports therapists’ decision-making and reduces bias between different treatments, ensuring a personalized and standardized approach.
- By comparing predictive performance with a decisions-tree based model, results demonstrate that the proposed system outperforms it, showing that kinematic and demographic data can be used to provide more accurate monitoring and predictions of rehabilitation outcomes throughout the robotic-assisted rehabilitation process.

## Results

This section presents the evaluation of the proposed models (LSTM models) alongside a conventional machine learning approach (optimized Random Forest models), yet state of the art in clinical data processing. Both models make use of the kinematic data handling methodology presented in this work, showing its applicability in practical contexts of predictive systems. The Random Forest for the first DST model was optimized with the following hyperparameters: 300 trees, 10 as the maximum depth of each tree, 5 as the minimum number of samples required to split an internal node, and 20 as the minimum number of samples required to be at a leaf node, while for the second DST the parameters were set to 350, 15, 5 and 5 respectively. All models were trained and evaluated on the exact same train/test folds to ensure a fair comparison. The LSTM models are described in detail in Sect. 4.5.

**Table 1:** Comparison of RMSE, MAE, Overall Accuracy (OA), and F1-Score between the proposed models and Random Forest. Where **CS**: Clinical Scores and where **DLP**: Difficulty Level Parameter.

Metrics	Proposed Model				Random Forest			
	Mean±Std	Min/Max	Mean±Std	Min/Max	Mean±Std	Min/Max	Mean±Std	Min/Max
**CS**	**RMSE**	**RMSE**	**MAE**	**MAE**	**RMSE**	**RMSE**	**MAE**	**MAE**
ARAT	$30.0\pm 6.9$	10.0/46.0	$23.0\pm 6.4$	8.0/40.0	$38.0\pm 6.8$	20.0/62.0	$31.0\pm 6.0$	16.0/56.0
FMA	$23.0\pm 5.6$	12.0/41.0	$18.0\pm 4.9$	7.4/30.0	$28.0\pm 5.1$	13.0/50.0	$22.0\pm 4.9$	10.0/45.0
MI	$20.0\pm 5.3$	6.0/35.0	$15.0\pm 4.2$	5.0/26.0	$24.0\pm 4.1$	13.0/38.0	$19.0\pm 3.4$	10.0/33.0
**DLP**	**OA**	**OA**	**F1-Score**	**F1-Score**	**OA**	**OA**	**F1-Score**	**F1-Score**
Weight	$81.7\pm 4.4$	67.9/91.3	$81.8\pm 4.3$	68.8/91.5	$76.3\pm 3.2$	66.3/86.8	$75.9\pm 3.4$	65.7/86.8
Stiffness	$78.5\pm 5.2$	62.5/87.9	$78.6\pm 5.3$	61.8/87.9	$59.5\pm 5.3$	47.7/70.2	$61.5\pm 5.0$	50.0/71.3
Viscosity	$96.0\pm 2.6$	86.0/99.7	$96.0\pm 2.6$	87.3/99.7	$96.0\pm 1.6$	92.2/99.1	$96.0\pm 1.6$	92.1/99.1

### DST: clinical outcome prediction model

**Fig. 2:**
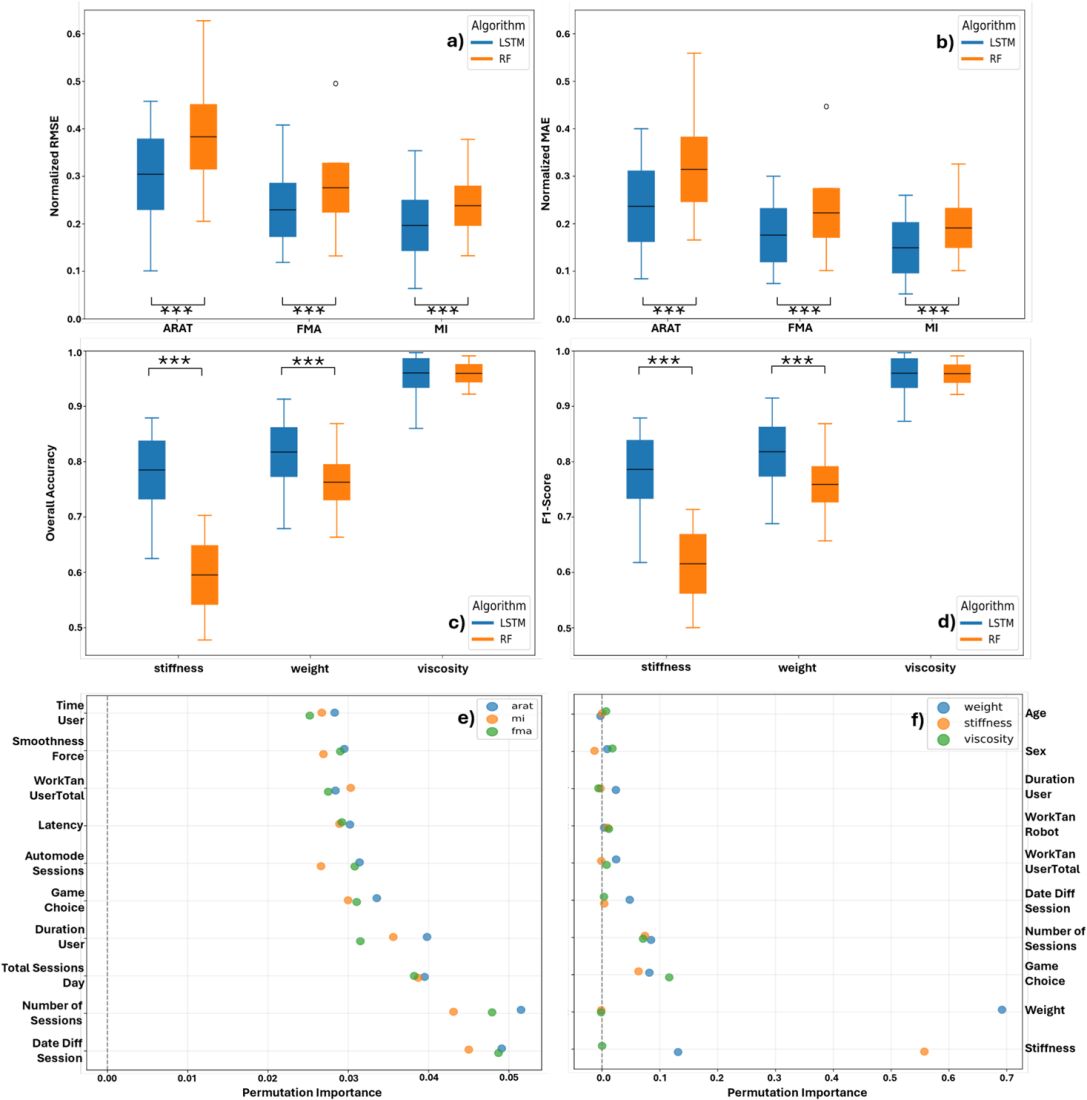
Boxplots of (**a**) the normalized RMSE and (**b**) the normalized MAE for the three clinical scores. (**c**) Boxplots of the overall accuracy and (d) the F1-score for the robot parameter suggestions. Each boxplot compares the proposed LSTM-based network with a Random Forest (RF)-based model. Asterisks (***) indicate a * p*-value less than 0.001. (**e**) and (**f**) show dotplots of the permutation importances for the 10 most prominent features.

The clinical outcome prediction model was evaluated using RMSE and MAE, with error calculations performed for each session of every patient. The final metrics are averaged across all patients and sessions. The boxplots shown in Fig. 2 demonstrate that the proposed model outperformed the Random Forest model in terms of both RMSE and MAE for all clinical scores. This highlights the efficacy of LSTM networks in handling Time-Series data where past information is crucial for making accurate predictions.

The proposed models achieved lower RMSE and MAE scores compared to RF for all three clinical scores (ARAT, FMA, and MI). The best performance was observed for the MI score, with a mean RMSE of $0.20\pm 0.053$ and mean MAE of $0.15\pm 0.042$ for the Stacked-LSTM model as seen in Table 1. These results confirm the hypothesis that LSTM networks, with their capacity to leverage time-dependent data, can provide superior predictive performance in clinical outcome forecasting when compared to conventional machine learning models like Random Forest.

This hypothesis is further supported by the permutation importance analysis shown in Fig. 2e, where the three most influential features are time-related. Notably, however, the overall contribution of individual features is relatively low, suggesting limited predictive power across the feature set.

**Fig. 3:**
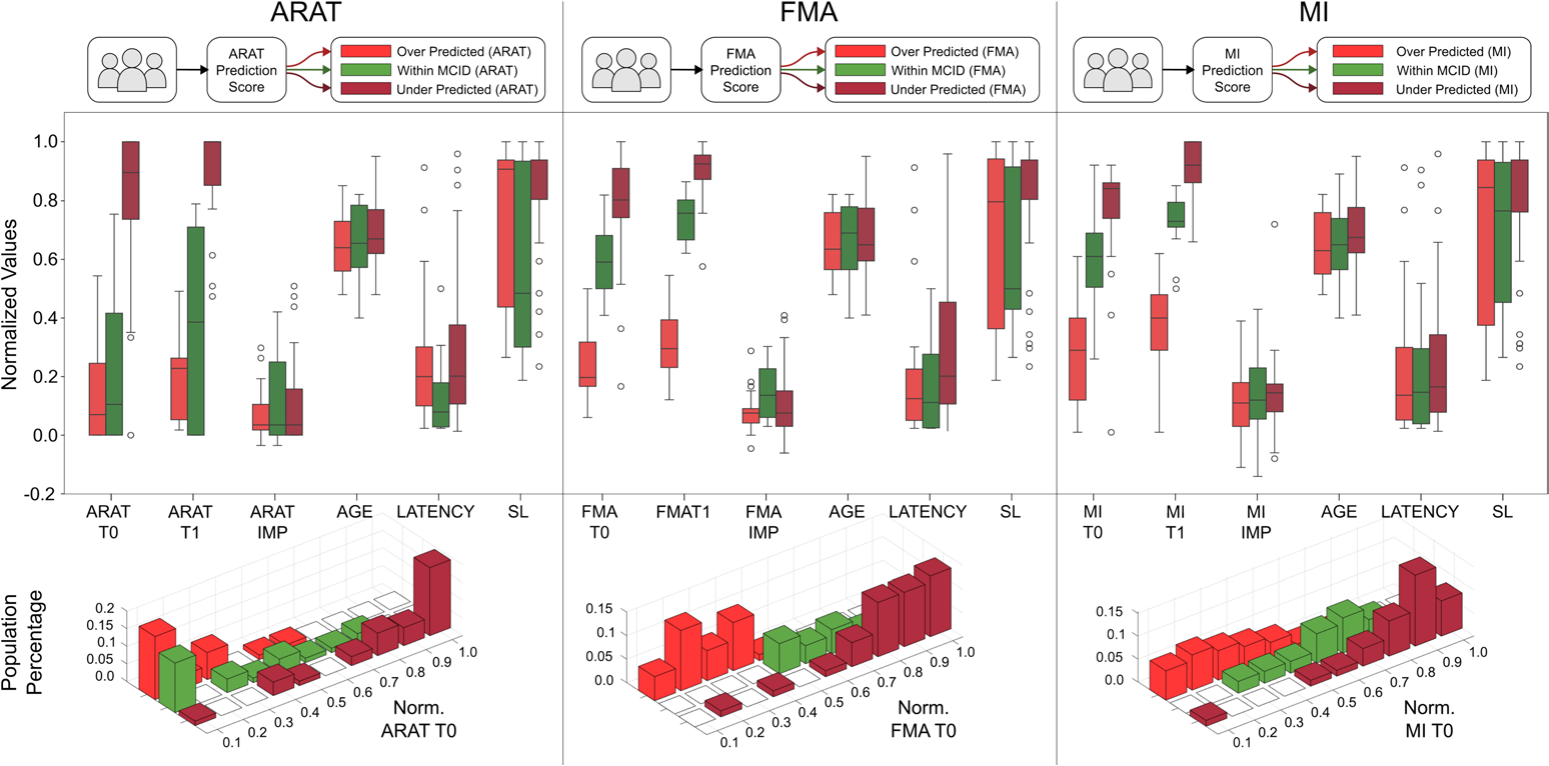
Post processing of the results. Patients are grouped based on the clinical outcome prediction error: green if within the Minimal Clinical Important Difference (MCID) range (between -10% and +10% PD), red if over-predicted, and dark red if under-predicted. Top graphs show groups with respect to clinical scales (pre-assessment ($T_0$), post-assessment ($T_1$), and delta ($T_1 - T_0$) on ARAT, FMA, and MI), demographics (age and latency), and data-related (SL, sequence length, i.e. the processed total number of days of therapy). Bottom graphs represent groups in a histogram of the whole population, based on clinical scale scores at $T_0$.

Further analysis on outcome prediction performance are provided in Fig. 3. Patients are split in three groups, for each clinical score, based on the final clinical outcome prediction error: green if it was within the Minimal Clinical Important Difference (MCID) range (between -10% and +10% PD), red if over-predicted (less than -10% PD), and dark red if under-predicted (greater than +10% PD). The figure shows how these population groups distributed with respect to initial clinical scales (top graphs in Fig. 3), and as a histogram of the whole population (bottom graphs).

### DST: Difficulty recommendation model

The evaluation of the difficulty recommendation models utilized Overall Accuracy (OA) and F1-Score metrics. Recommendations were based on actual parameters used by therapists throughout the study.

By investigating the boxplots in Fig. 2, we notice that the proposed models consistently perform on average above 85% for both metrics. This demonstrates their effectiveness in mimicking therapist decisions. The RF models, on the other hand, showed a lesser performance in particular for the stiffness parameter, with an almost 20% lower performance than the proposed model. Regarding the viscosity parameter, the mean OA and F1-Score were comparable between the proposed models and the RF.

The precise evaluation numbers can be seen in Table 1, where the proposed models achieved an average OA of 81.7% for weight, 78.5% for stiffness, and 96.0% for viscosity, with corresponding F1-Scores of 81.8%, 78.6%, and 96.0%, respectively.

In addition, analysis of the permutation importances illustrated as dotplots in Fig. 2f reveals that the most influential features are primarily related to the robot parameters themselves. Specifically, the weight feature in the weight model (importance score of 0.69) and the stiffness feature in the stiffness model (importance score of 0.55), as these are highly correlated with the respective outputs. Other notable contributors include the Game Choice (averaging approximately 10% importance across all three models) and various time-related features.

## Discussion

A methodology for structuring a kinematic data based dataset, to be used as input source for deep-learning processing, has been presented in this work, alongside with an experimental application of the method with an LSTM-based network and a large robotic rehabilitation dataset. The possibility to rely on time-sequences and time-related features has shown promise in improving the performance of predictive algorithms for post-stroke rehabilitation therapies, both for predicting the clinical outcomes, and for suggesting optimal parameters to be set for the robotic-assisted exercises.

The LSTM-based network, trained to make a better use of the data handling methodology proposed in this work, revealed to perform better than a decision-tree based model (see Fig. 2). This means that, adopting the proposed method can be beneficial if coupled with a proper model. Most of the studies in literature focus on making a single prediction on a specific clinical scale using an input set mainly composed by clinical scales themselves, demographic data, and MRI images^30^. Many studies focused, also, on making classifications on the Modified Rankin Scale (mRS) separating favorable outcomes from non-favorable ones^31,32^. In some cases, the National Institute of Health Stroke Scale (NIHSS) is used as a target for classification systems to aid therapists in decision making^33^. Given the relatively low amount of data to process and the inherited rule-based nature of prognostic tools from traditional approaches, it is very common to find decision-tree based techniques in the state of the art^22,34^. The limitation of this approach is in both the fixed time-point of the prediction (typically a single prediction right after the clinical pre-assessment)^9^, caused by the absence of time-based features in the input set, and the difficulty in handling Time-Series with these algorithms. Random forest is one representative of this algorithms family, also used in previous works^8,35^, but at the same time it produces very similar outcomes to other decision-tree based algorithms which makes the current comparison robust. The proposed methodology and consequent test architecture are able to leverage the variability of data collected over time and to build predictions at a variable time-point, also better showing the influence of time-varying metrics in robotics rehabilitation settings.

We further investigated potential factors related to the prediction performance. The analysis depicted in Fig. 3 evidences the distributions of several clinical and demographic variables, after grouping the whole population based on the performance of the prediction: a prediction hereby, is considered good (green group) if the prediction error falls within the MCID range, otherwise is considered bad (red groups, either overpredicting or underpredicting). It has to be noted that in this case the MCID is used as a way to assess the goodness of a prediction: if a prediction is within the MCID it means that the prediction error is low enough not to cause a different approach by clinicians in the design of the therapy. This aspect is key to understand the clinical acceptability of a prediction error: it does not aim at being zero but rather below the threshold of clinical relevance. In literature, there are some studies analyzing the MCID or assessing the average of improvements on well-known clinical scales. Lin et al. reported a value of 15 points as the average improvement on 69 post-stroke patients on the Motricity Index and a MCID of 13 points^36^. Aprile et al. reported an average improvement of 17.4 points on Motricity Index, 8.5 points on FMA, and 6.0 points on ARAT, on a sample of 91 acute and sub-acute post-stroke patients undergoing robotic rehabilitation treatment^37^ while Narayan assessed a MCID for FMA equal to 9-10 points on 71 sub-acute post-stroke patients^38^. Lang et al. found out a MCID on the ARAT clinical scale from 12 to 17 points, depending on the side of the lesion (i.e. dominant or non-dominant arm) on a sample of 52 sub-acute post-stroke patients^39^. Giving these variabilities in clinical scales and for simplicity of the analysis, the MCID has been set to the 10% of each scale.

The immediate information based on Fig. 3 is that patients with lower starting clinical scores (which also correlated to lower clinical scores at discharge) are over-predicted. This result is also visible in the histograms, noting that the population of the over-predicted patients is more present to the lower clinical scale. On the other hand, patients with higher starting clinical scores are usually under-predicted. Taking into consideration also the histograms, it is clearly visible that the population of this group tends to be on the higher clinical scale site. This particular behaviour can be noted on FMA and MI scales, which also correlate each other, but not on the ARAT. Regarding the patient improvements on all three clinical scales, we notice a strong pattern, where patients with greater clinical improvement are usually found within the MCID range. Considering the patient’s age there is no strong evidence between the groups and clinical scales. Regarding the latency, which is the delay between the first stroke event and the first rehabilitation session, we notice that for ARAT and FMA, patients with lower latency tend to be in the within MCID range, while the same statement cannot be made for the MI score. Another interesting fact is, that patients who have completed the most sessions (longest Sequence Lengths) are also most of the time under-predicted. The final observation made from Fig. 3 is deriving again from the histograms, there we noticed that the population based on FMA and MI are equally split, showing the over predicted patient up until the 45% mark, then having the within MCID patients close to the 85% mark, and finally having the under predicted patients. For the ARAT population this pattern changes, with overlap between the over-predicted and within-MCID groups. Here however, the histogram of the whole population for the ARAT scale appears concentrated at the two limits of the scale. Information shown in the bottom plot of Fig. 3, ensures that under-predictions and over-predictions do not depend on unbalanced composition of datasets but they may depend on hidden correlations between kinematic data and clinical scale scores.

Regarding the Difficulty Recommendation tool, the obtained results show the proposed approach can achieve overall better performance than conventional CDSS. In particular, the LSTM approach obtained comparable results to the RF for the Viscosity parameter, and a noticeably better prediction for the Weight and Stiffness parameters. Beyond the proposed comparison with a state of the art method, results are also informative about feasibility of DSS system based on similar robotic assisted scenarios, where kinematic data are continuously measured and made available throughout the rehabilitation therapy. The mean OA accuracy between the three parameters was 85,4%, and again 85,4% for the F1-Score, showing the capability of the method to provide updates of the exercise parameters close to the one actually chosen by an expert therapist in each recorded session. On one hand, this aspect is notably important, since it can also support the training of new clinical personnel that may not be expert of a certain rehabilitation device. On the other hand, it has to be noted that these suggestions are not necessarily linked to an improved motor recovery, but rather aim at reproducing an expert therapist’s behaviour. Given the high variability of clinicians’ choices on setting robot parameters, due to organizational factors alongside clinicians’ characteristics^40^, understanding how these ones can influence the recovery process may be hard and tedious. Using the proposed DST may be useful to define a more standardized behaviour, based on the kinematic features of patients’ movements, and to speed up the analysis of their effects on the rehabilitation pathway. The provided suggestions could be used together with more classic infotainment system, adopted as decision making support tools for clinicians^41^; these, typically provide information about physiological movement curves, serious game scores and similar outcomes. Adaptive robotic systems can also be configured as decision support tools able to change the way the system itself interacts with patients^42^.

To the authors’ knowledge this is the first attempt in literature to produce a CDSS in the robotic rehabilitation domain, mainly based on kinematic data. The results obtained in this study are expected to impact the way data coming from rehabilitation robots can be used, also considering the increasing adoption of these systems in clinical settings. This approach offers a viable compromise between automated and human-selected recommendations for adapting the exercise parameters to the progress of the patient. Compared to post-stroke and machine learning studies found in literature, with a broader use of more simple and explainable models like linear regressions and decision trees, this study aims to provide evidence and support about the proper handling of temporal information in deep-learning approaches, and their potential in providing clinical outcome predictions and assistance for therapists.

This work is primarily limited by the sample size, which may reduce the statistical power of the analysis. Additionally, the exclusion of patients with a low number of recorded sessions inherently narrows the applicability of the system, potentially limiting its generalizability across the full spectrum of patient profiles. Another limitation is that robot parameters suggestions are not based on motor recovery but on previous expert clinicians’ choices, basically orienting the DSS to reproduce their experience instead of optimizing its efficacy on the motor recovery. This last task remains an open challenge that will be specifically approached in future studies. In addition, even with predictions and suggestions available, therapists may still face challenges in effectively managing robot-assisted therapy due to the lack of widespread training and standardization in robotic rehabilitation. Furthermore, incorporation of this CDSS into clinical workflows should prioritize transparency and therapist control. Although automation bias is a known risk in clinical decision support systems, in our setup we explicitly avoided persuasive messaging and allowed the therapist to retain full control by presenting predictions and suggestions without prescriptive language. A user interface could further mitigate this risk and support clear communication of the system’s supportive, rather than directive, role. It should also be noted that the robot used in the study is a planar commercial device operating with a fixed set of serious games. These games share similar kinematic logic, focused on applying forces and guiding movement, which may limit the variability of kinematic data collected. This, in turn, may impact the richness of the model’s training. Nonetheless, this method is believed to be adaptable to more advanced contexts, such as exoskeletal systems, which still rely on time-series kinematic data but might yield different predictive performances.

From a clinical integration standpoint, implementation of this system in real-world practice still faces challenges. The interpretability of machine learning predictions for therapists, and the need for seamless integration into clinical software and workflows are all current limitations. For example, modern Large Language Models could be used to provide a full description of the predictions’ and suggestions’ rationale and motivation, thus, supporting in a proper way the clinicians that, in any case, must always be at the center of the design of these systems. Further development should focus on clinical validation, interface usability, and regulatory compliance to facilitate real-world deployment. Beside this, it is indeed important that CDSSs in the future provide informed suggestions but always leaving clinicians the freedom to have the last word. From a regulatory point of view, it is crucial understanding who is responsible for data and which entity owns the predictive model. Clinics already treat patients’ personal data (procedures and documentation can be slightly different from country to country) thus the implementation of predictive models in the clinical practice could be put in the same domain, not triggering major issues in this sense. A more delicate case occurs if the producers of rehabilitation robots also own predictive models, as part of additional and advanced modules of their products, thus requiring these systems to be compliant to the GDPR policies and/or the new incoming AI Act.

Finally, the performance of the predictor on the test set revealed a median prediction error above the MCID threshold for the three clinical scales. The proposed approach opened new ways to include, interpret, and benefit from kinematic data in future, more complex solutions, but it focused on kinematic data only as a specific use case. All clinical settings found in literature have clinical scale scores from initial assessments that can be used in the input set as well, adding benefits in lowering prediction errors and bringing them within satisfying ranges.

## Methods

### System architecture

This section outlines the adopted system architecture (Fig. 1), starting with Data Collection, which includes initial clinical assessments and robot-aided rehabilitation sessions via virtual serious games. Three data types are collected: demographic, clinical, and kinematic. Next, data processing steps prepare the training and testing datasets, both derived from the same source data. These datasets are then fed into two separate networks that together form the Clinical Decision Support System. Each step is detailed in the following sub-sections.

### Data collection

**Fig. 4:**
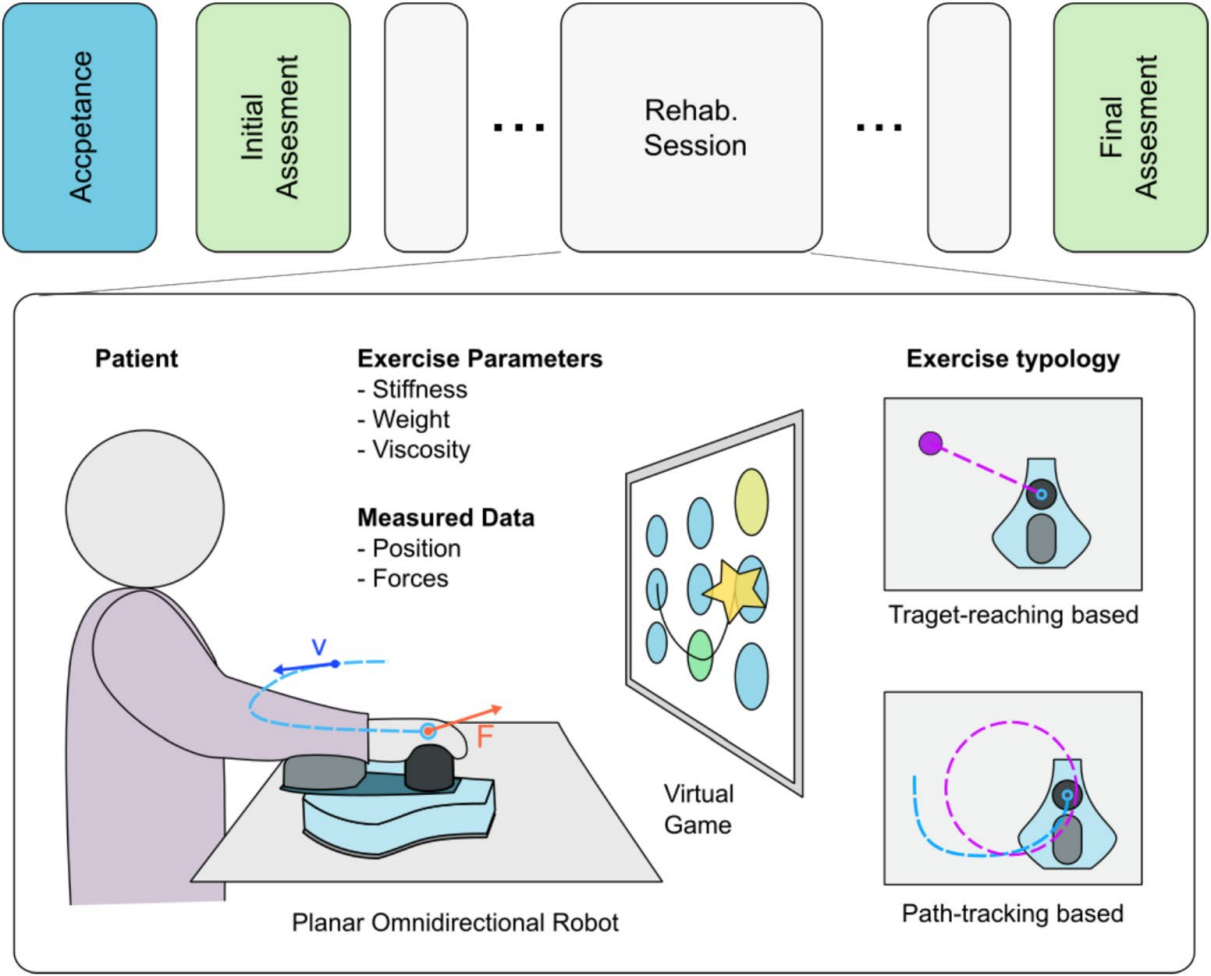
Data collection protocol and detail of the clinical robotic rehabilitation setup involving virtual exercises performed with robotic assistance.

The dataset comprises recordings from 121 patients who underwent rehabilitation therapy with the MOTORE robot, combining data from two previous studies registered at clinicaltrials.gov (NCT02879279^8,37^ and NCT05238389^35^). For the former, the study was approved by the Ethics Committee of Fondazione Don Carlo Gnocchi on April 6, 2016 (FDG_6.4.2016), for the latter (“iMotore” study) the study was approved by the Ethics Committee Lazio 1 on December 29, 2021 (STS CE Lazio 1/N-873). The therapy sessions varied in number and involved upper-limb planar motor tasks with modulated robotic assistance using the MOTORE system with serious games (Fig. 4). Robot parameters have been set session-by-session by expert clinical personnel, with a previous well-established experienced with the MOTORE robot.

The dataset includes demographic, clinical, and kinematic information. Demographics cover age, gender, and latency (days from stroke to therapy start). Clinical data, collected via pre- and post-treatment assessments, include the Fugl-Meyer Assessment ($\text {FMA} \in [0, 66]$), Action Research Arm Test ($\text {ARAT} \in [0, 57]$), and Motricity Index ($\text {MI} \in [0, 100]$). The post-treatment clinical scores are used to calculate prediction errors between actual and predicted outcomes. The robot-derived data consists of high-level features extracted from force and position data throughout the rehabilitation session between the interaction of the patient and a list of virtual serious games.

For the rehabilitation session by itself, therapists must select a serious game and define its difficulty by configuring three robot parameters: stiffness, weight, and viscosity. Each parameter has predefined levels: stiffness and weight can be set to Low, Mid, or High, while viscosity has two levels: Low and High. The robot parameters are defined in Table 2. The choices of the parameters made by the therapists are set as ground truth for the recommendation model presented in 4.5. Thus, the recommendations are based on expert human knowledge. Once the difficulty level is set, patients play various serious games while the robot monitors movement and applies forces. Therapists can choose from seven games. Playing different games in a session offers dual benefits: it reduces task repetition, enhancing patient engagement, and provides unstructured temporal data, which, after processing, LSTM networks utilize effectively. In cases of motor incapability, serious games can be completed in automode, where the patient’s arm is moved passively, resulting in limited data value for our models. Consequently, automode sessions were excluded from the analysis due to the absence of patient’s active motion information but keeping track of their number in the input set.

### Data processing

This chapter explains the processing steps applied to the therapy data collected. We also present our novel method for creating multidimensional Time-Series dataframes for each patient, integrating demographic data with kinematic and time-related features from serious game sessions.

#### Timeline creation, noise reduction and time-Series filtering

The robot generates high-level kinematic features for each successfully completed serious game, deriving from the position and force measurements. Since most games are played multiple times per session, we reduced noise by averaging the kinematic features for the same game on the same date. These averaged game rounds are termed “sessions” and represent a Time-Step per day. Since not all serious games share the same high-level features, we considered only those features with a mutuality above 70%. Missing values were filled using the average for the specific date. If no other values were available for that date, a front-fill operation was applied. By organizing these sessions in chronological order, we construct each patient’s Time-Series. Multiple games played on the same day can result in Time-Steps with temporal features from the same date. These procedures are important as our proposed networks highly benefit from structured temporal information. To maintain dataset consistency, two exclusion criteria were applied: a minimum of 10 different attendances per patient and the requirement of completing post-treatment clinical assessments. These criteria led to the exclusion of 36% of patients (43 out of 121), resulting in a final dataset of 78 patients. Among the excluded individuals, 19 did not complete the rehabilitation therapy and therefore lacked post-treatment assessments. The remaining 24 patients, although they completed the therapy, attended fewer than 10 distinct rehabilitation sessions and were also excluded.

#### Features extraction

At this stage, each patient’s data includes demographic and kinematic features. To enhance the dataset, we also add time-related features, which refer to the game’s completion date and the number of completed games on the same date. The Time-Related features consist of seven features, as noted in Table 2.

In addition, we enhance the kinematic feature group with the robot parameters, which include weight, stiffness, and viscosity values set by the therapist for each game. When multiple games are averaged into a session, these parameters are assigned to the nearest available value.

**Table 2:** Demographic, Kinematic, and Time-Related Features.

Set of Features	Feature Name	Description
Demographics	Age	The age of the patient at the start of the therapy
	Gender	The gender of the patient
	Latency	The latency, in days, between the stroke and the start of the rehabilitation process
Kinematic Related Features	userVelocityAvg	Average speed of the robot during the whole recorded session
	workUserTotal	Total work done by the user while pushing the robot in all directions
	workTanUserTotal	Component of the total work done along the direction demanded by the game
	precisionWork	The definition of the ratio between workTanUserTotal and workUserTotal
	distanceTask	Nominal length completed by the patient based on the specific exercise
	autonomyDuration	Percentage of time that was spent in self-mode and the robot was moving compared to the total time that the robot was moving
	velocityNorm	Average speed of the robot divided by the typical mean value of a healthy subject
	durationUser	The total duration to complete the exercise
	workTanRobot	Estimated work done by the robot to assist the patient along the trajectory
	params	The timerate of the precisionWork parameter
	workTanUserSelf	Work done by the patient along the trajectory in self-mode
	overall	Weighted final score for the exercise, considering time, speed, and work
	calories	Estimation of user energy consumption during the exercise
	timeUser	Duration of the game played in seconds
	smoothness_force and smoothness_kin	Smoothness of force and position recordings, computed using the Spectral Arc Length algorithm
	stiffness, weight and viscosity	Stiffness determines the force the robot applies in response to displacement from the reference trajectory. Weight simulates mass and inertial forces at the interaction point. Viscosity represents resistance proportional to the velocity of movement, similar to moving through a fluid.
	Game Choice	The selection between seven games (one-hot encoded)
Time-Related Features	date_diff	The difference in days between the current session and the first session
	date_diff_session	The difference in days between session dates
	remaining_day_of_therapy	A declining counter based on the timesteps of each patient
	number_of_sessions	The number of sessions from a specific game completed in a day
	total_sessions_day	The total number of all game sessions completed in a day
	total_sessions_incr	An increment counter of all sessions completed up to this date
	automode_sessions	The number of sessions completed in automode (no user effort)

#### Data normalization

To prevent features with large ranges from dominating the model, all features were normalized to a range between 0 and 1. Demographic features (Age, Gender, Latency) were adjusted by dividing with the maximum, while for the latency, first we applied a logarithmic scale and then divided by its maximum value for all patients. Time-Related features were normalized per patient, based on their actual deviation from the planned rehabilitation schedule. Normalization was performed using the maximum value of each Time-Related feature for each patient, reflecting the patient’s unique progress during rehabilitation. Kinematic features, including robot parameters, were normalized to the 0–1 range. For kinematic features, this was done for each game scenario. Robot parameters (weight, stiffness, and viscosity) were normalized by dividing each parameter by its maximum value. This procedure ensured that higher-performing patients set the maximum values for each game feature, maintaining a natural reference for comparison and consistent performance scaling.

#### Padding or shrinking

**Fig. 5:**
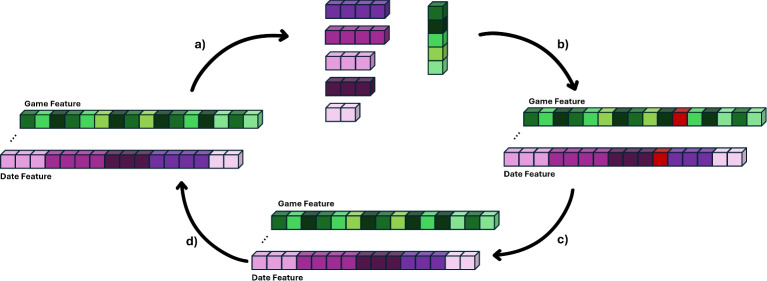
The proposed algorithm dynamically shrinks time-series data to a desired length through iterative grouping and deletion. (**a**) The sequence is separated based on dates, and the popularity of the resulting groups is calculated. (**b**) The session is identified from the longest group, selecting the most prominent game. (**c**) This session is then removed from the dataset. (**d**) After each deletion, the algorithm dynamically recomputes the groups and their popularity, iterating the process until the sequence reaches the required length.

To create a homogeneous dataset, we ensured that all patients’ Time Series were of equal length. Depending on the original length, we applied either padding or shrinking to reach a target of 64 Time Points. We selected 64 Time Points because most Time Series naturally clustered around this length. Additionally, our model architecture required a fixed input size. Importantly, individual game sessions were not critical for prediction accuracy. Therefore, for longer Time Series, we removed sessions from days with a high number of game sessions to meet the target length. This improved computational efficiency without negatively affecting model performance. Time-Series with fewer than 64 Time-Points were padded with zeros at the end of the Time-Series, and the non-informative zeros at end of the Time-Series were masked out by the network during training and evaluation. Considering the shrinking of the Time-Series, a more complex approach was implemented for a Time-Series length greater than 64 Time-Points. This shrinking method begins by creating groups based on Time-Points from the same date, as these Time-Points may contain multiple game sessions. The groups were then sorted in descending order based on the number of unique games played within the date group. When groups had the same length, they were ordered based on the minimum distance between adjacent groups. Next, a list was created, tracking the frequency of completed games for each patient, indicating the popularity of the games in each Time-Series. The final step involved identifying the most popular game from the group with the highest number of games completed on the same date and removing it from the Time-Series. After removing a specific game session, the algorithm dynamically recomputed the prementioned steps to maintain balance in the sparsity of the temporal dimension and the diversity of the completed games. An illustration of the proposed algorithm can be seen in Fig. 5.

### Training and evaluation

**Fig. 6:**
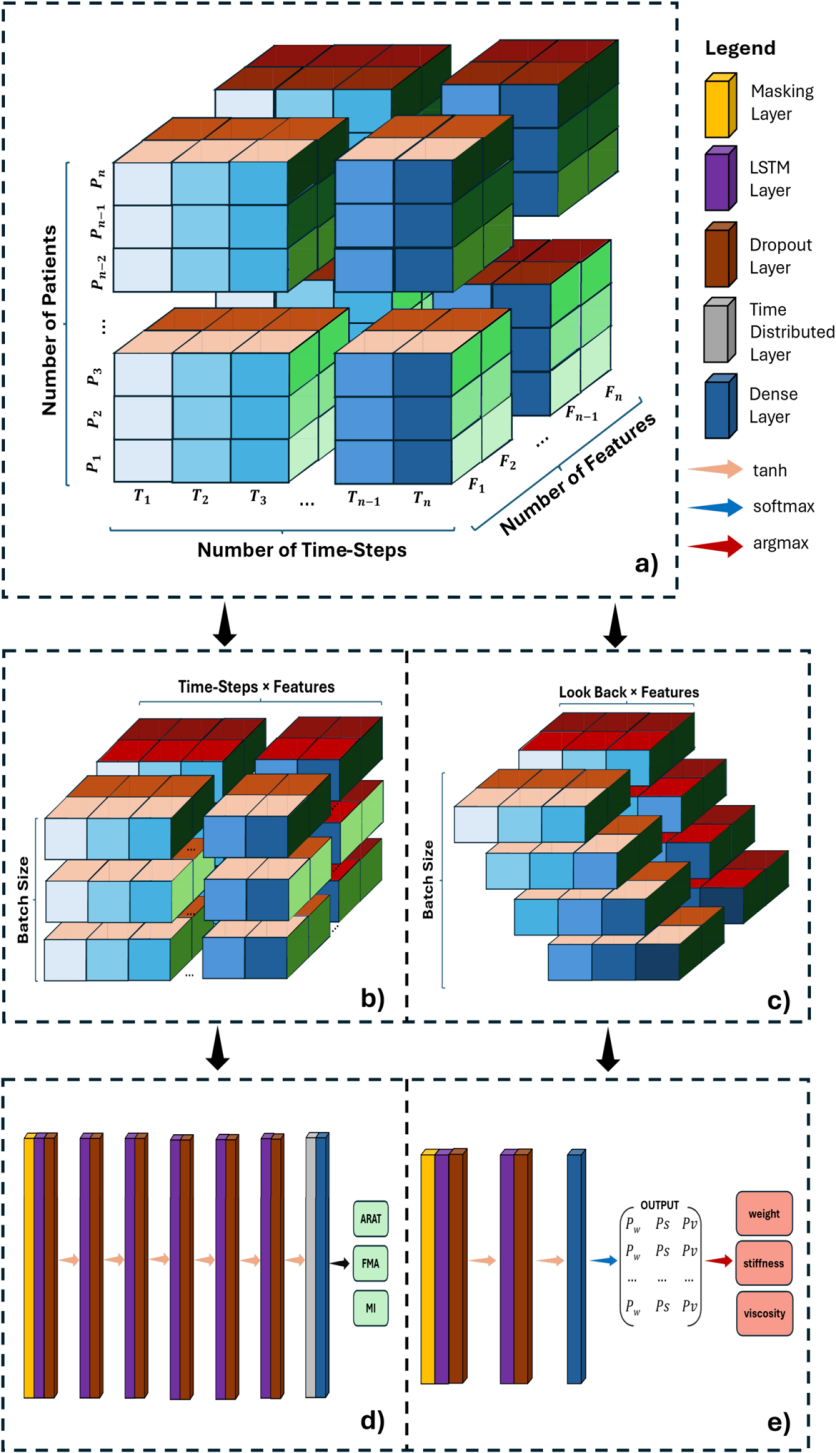
3-D illustration of (**a**) the data structure, (**b**) the input shape of the clinical outcome prediction model, and (**c**) the input shape of the robot parameter recommendation model. Each color palette on all three dimensions indicates a physical quantity. The blue color palette corresponds to different Time-Points; the red color palette corresponds to features; and the green color palette corresponds to patients. (**d**) The proposed architecture of the clinical outcome prediction DST and (**e**) the proposed architecture of the difficulty recommendation DST.

After completing all processing steps and concatenating all available data groups, we obtained the following data structure: $Patients \times TimeSteps \times Features$ Fig. 6a. From this data structure, we created the training and testing inputs for both Decision Support Tools (DSTs), which are introduced in Sect. 4.5. The data structure was reshaped so that the input corresponds to the problem we aimed to solve: providing clinical outcome predictions for each patient or recommending robot parameter setups for the next game session.

The first DST was trained at the patient level, involving a total of 78 samples. Consequently, the input shape for the clinical outcome prediction model is $BatchSize \times TimeSteps \times Features$ (Fig. 6b). This structure benefits the model by training on complete Time-Series data for each individual patient. After one-hot encoding the serious games, the feature dimension totals 36 features, with a detailed exaplanation of each feature presented in Table 2. The second DST provides recommendations for the three robot parameters to assist therapists. This model must make short-term recommendations based on limited prior information. Thus, we adapted the structure to $BatchSize \times LookBack \times Features$ (Fig. 6c), where *LookBack* represents the number of previous Time-Points needed for generating recommendations.

### Clinical decision support system

This section introduces our proposed clinical outcome prediction and robot parameter recommendation models, both integral to our decision support system. The prediction model forecasts clinical scores throughout the therapy, while the recommendation model assists by suggesting appropriate difficulty levels for rehabilitation sessions. Regarding the clinical outcome prediction model, the three clinical scores at discharge (ARAT, FMA and MI) are used as ground truth labels throughout the analysis, and the RMSE (1) and MAE (2) errors are calculated between the predicted clinical values and the actual discharge values. In contrast, the ground truth labels for the recommendation model are the three robot parameters of the next session (stiffness, weight, and viscosity), originally selected by expert therapists, and the OA (3) and F1-Score (4) are calculated based on the predicted and actual values.

Both models were implemented using Python with the TensorFlow Keras library^43^, supported by Pandas and NumPy for data processing. Parameter optimization was conducted using the ParameterGrid function from the scikit-learn library, which also provided Random Forest (RF) implementations for baseline comparisons in regression and classification tasks. All training and evaluations were performed on an NVIDIA GeForce RTX 4080 GPU with 16GB VRAM and an Intel Core i7 CPU.

#### Decision support tool: clinical outcome prediction

To deliver real-time, personalized clinical outcome assessments, we utilized an LSTM network with a TimeDistributed layer combined with a Dense layer. This setup processes data per Time-Step rather than per batch. Extensive hyperparameter tuning was conducted to optimize the network’s configuration. A parameter grid search identified an optimal architecture of six LSTM layers, each with 32 cell units and tanh activation functions. A 20% Dropout layer was added after each LSTM layer, with the initial learning rate set to 0.1 and RMSprop used for the loss function. A batch size of 1 was found to yield the best results, allowing for patient-by-patient training. The model architecture is shown in Fig. 6d.

#### Decision support tool: difficulty level recommendation

The second DST consists of three separate models for recommending the robot parameters: stiffness, weight, and viscosity, which the combination declares the difficulty of the serious game. For stiffness, a stacked-LSTM with two layers was used, while simpler LSTM architectures were more effective for weight and viscosity. Hyperparameter tuning was performed for all models, with chosen parameters summarized in Table 3.

**Table 3:** Model Parameters for: Stiffness, Weight, and Viscosity.

Parameter Name	Stiffness Model	Weight Model	Viscosity Model
Cell Units per LSTM layer	64	32	64
Number of LSTM Layers	2	1	1
Look Back	3	3	5
Dropout Rate after each LSTM Layer	0.1	0.2	0.2

All models used tanh as the activation function and adam for the loss function, with a batch size of 256 yielding optimal accuracy. The architecture of the stiffness model is shown in Fig. 6e. The weight and viscosity models do not include the second LSTM layer. Unlike the clinical outcome model, these models output class probabilities. The most probable class is selected using an argmax operation and mapped to the corresponding parameter value.

#### Evaluation metrics

The evaluation of the proposed clinical outcome prediction tool occurred with two error calculation metrics. The first metric is the root-mean-square-error (RMSE), equation (1) which is calculated by taking the square root of the average of the squared differences between the true and predicted values. The second metric used in the evaluation process is the mean-absolute-error (MAE), equation (2). The Mean Absolute Error is the average of the absolute differences between the true values and the predicted values.

1 $$\begin{aligned} \text {RMSE}&= \sqrt{\frac{1}{n} \sum _{i=1}^{n} \left( y_i - \hat{y}_i \right) ^2} \end{aligned}$$

2 $$\begin{aligned} \text {MAE}&= \frac{1}{n} \sum _{i=1}^{n} \left| y_i - \hat{y}_i \right| \end{aligned}$$

where:

- *n*: The total number of data points.
- $y_i$: The true value at the *i*-th point.
- $\hat{y}_i$: The predicted value at the *i*-th point.

Both metrics provide a measure of error in the same unit as the target variable, and therefore resulting easier to be interpreted. The RMSE is more robust to outliers, while the MAE provides an intuitive understanding of the typical difference between predicted and actual values. Regarding the evaluation of the second DST, the model recommending the difficulty level of the exercise, two other metrics were used. The first metric is the Overall Accuracy (OA) (Equation 3) computed as the ratio of correctly classified cases over the total number of cases. The second metric is the F1-Score (Equation 4) computed as the harmonic mean of precision and recall, which is especially useful in unbalanced datasets.

3 $$\begin{aligned} \text {OA}&= \frac{TP + TN}{TP + TN + FP + FN} \end{aligned}$$

4 $$\begin{aligned} \text {F1-Score}&= \frac{2 \cdot \text {Precision} \cdot \text {Recall}}{\text {Precision} + \text {Recall}} \end{aligned}$$

where:

- *TP*: True Positives (correctly predicted positive cases).
- *TN*: True Negatives (correctly predicted negative cases).
- *FP*: False Positives (incorrectly predicted as positive cases).
- *FN*: False Negatives (incorrectly predicted as negative cases).
- $\text {Precision} = \frac{TP}{TP + FP}$: The proportion of true positives among all positive predictions.
- $\text {Recall} = \frac{TP}{TP + FN}$: The proportion of true positives among all actual positive cases.

One last metric implemented for the post-process analysis is the percentage difference (PD) as calculated in Equation 5.

5 $$\begin{aligned} \text {PD}&= \frac{y - \hat{y}}{\text {max\_score}} \end{aligned}$$

where:

- *y*: The clinical outcome at discharge, representing the true label.
- $\hat{y}$: The mean predicted value for the specific patient.
- $\text {max\_score}$: The maximum achievable score on the clinical scale.

For this reason, positive values of PD indicate predictions under the actual value, while negative values of PD indicate predictions over the actual value.

#### Feature explainability

To assess the relative contribution of each input feature to the model’s predictions, we applied Permutation Feature Importance (PFI) analysis. This technique evaluates the importance of each feature by measuring the increase in prediction error after randomly shuffling its values, thereby breaking the relationship between the feature and the target. In our implementation, we computed a baseline score using the root mean square error (RMSE) or accuracy, depending on the prediction task. Then, for each feature, we permuted its values across the validation data and recalculated the model’s performance. The difference between the baseline and the perturbed scores quantifies the feature’s permutation importance. We performed the analysis for the two proposed models: (1) clinical outcome scores (ARAT, FMA, MI), using RMSE as the metric, and (2) robot parameter prediction (stiffness, weight, viscosity), using classification accuracy. Importance scores were normalized to sum to one, and results were visualized as dotplots to highlight the most influential features in each task.

## Supplementary Information

Supplementary Information 1.

## Data Availability

The datasets generated during and/or analysed during the current study are available from the corresponding author on reasonable request.
