# Supplementary figures and images for: Systematic data management for effective AI-driven decision support systems in robotic rehabilitation
Source: Sci Rep. 2025 Jul 30;15:27835. doi: 10.1038/s41598-025-09740-2 (PMC12310958; doi:10.1038/s41598-025-09740-2)

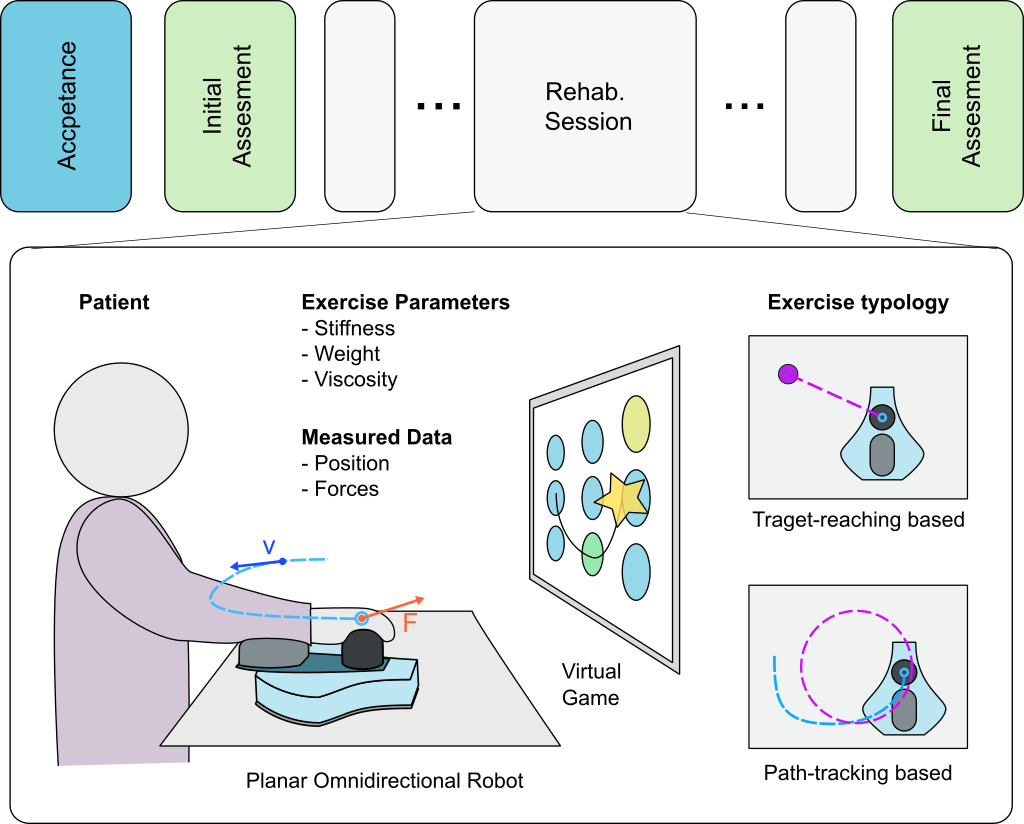

Supplement: Supplementary file 1 — Supplementary Information 1. [file 41598_2025_9740_MOESM1_ESM.png]
